# 

**Published:** 1897-08-21

**Authors:** 


CADBURY'S COCOA

"The standard of highest purity at present attainable."—*Lancet.*

CADBURY'S COCOA

NO ALKALIES USED.

### THE HOSPITAL

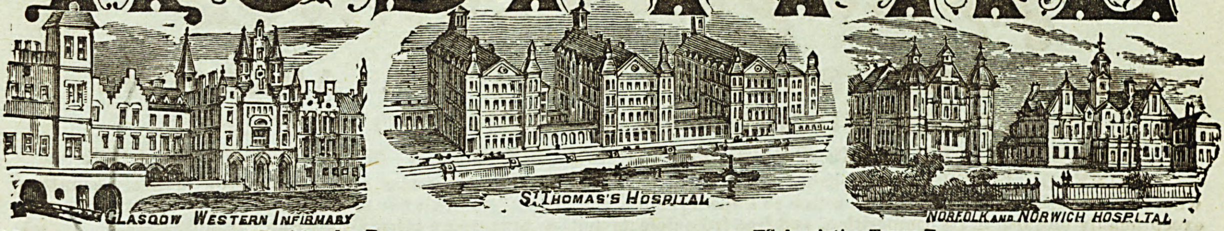

No. 569. Vol. XXII. [Registered as a Newspaper.] SATURDAY, AUGUST 21ST, 1897. [Subscription Terms, see p. 350.] PRICE TWOPENCE.

